# Posterior Capsulorhexis as a Preventive Strategy: A Case of Bilateral Congenital Cataract With Divergent Outcomes

**DOI:** 10.7759/cureus.91325

**Published:** 2025-08-31

**Authors:** Grigor Kamushadze, Davit Shengelia, Gigi Gorgadze, Bacho Shengelia, Saiali Ibragimova

**Affiliations:** 1 Department of Ophthalmology, Clinic "New Hospitals", Tbilisi, GEO; 2 Department of Ophthalmology, Tbilisi State Medical University, Tbilisi, GEO; 3 Faculty of Medicine, Tbilisi State Medical University, Tbilisi, GEO; 4 Ophthalmology, Givi Zhvania Pediatric University Clinic of Tbilisi State Medical University, Tbilisi, GEO

**Keywords:** cataract surgery, nd:yag laser capsulotomy, pco prevention, posterior capsule opacification, posterior capsulorhexis

## Abstract

Posterior capsule opacification (PCO) is a common delayed complication of cataract surgery, particularly in younger patients and those receiving multifocal intraocular lenses (IOLs). Intraoperative posterior capsulorhexis has been proposed as a preventive technique, but its use in adult cataracts is less documented. A 27-year-old patient presented with bilateral congenital posterior subcapsular cataracts, high myopia, and astigmatism. Comprehensive preoperative examination, including visual function and retinal imaging, was performed. The patient underwent phacoemulsification with toric multifocal IOL implantation in both eyes. Intraoperative posterior capsulorhexis was performed in the right eye, while the left eye underwent standard surgery without capsulorhexis. One month postoperatively, both eyes achieved excellent visual acuity (OD: 1.0, OS: 1.0) and satisfactory refractive outcomes. Four months postoperatively, the left eye developed PCO, necessitating Nd:YAG laser capsulotomy, after which the patient experienced retinal detachment requiring vitrectomy. Vision in the left eye decreased to 0.6. The right eye, which underwent posterior capsulorhexis, remained free of PCO and complications, with stable visual acuity of 1.0. This case highlights the potential benefit of intraoperative posterior capsulorhexis in preventing PCO and secondary complications in high-risk adult patients undergoing cataract surgery with multifocal IOLs. Posterior capsulorhexis may reduce the need for Nd:YAG laser capsulotomy and its associated risks, particularly in young adults with congenital cataracts and high myopia.

## Introduction

Cataract - a clouding of the natural lens of the eye - is a leading cause of visual impairment and blindness worldwide, especially in developing regions. Based on available data, cataracts are most common in the white American population, where the prevalence is 17-18 individuals per 100 people [1]. The disease most often appears in the fifth and sixth decades of life and is 1.3 times more common in women than in men. The disease progresses gradually and is the most common cause of blindness in the world (with the highest rate of blindness in Southeast Asia), thereby having a significant impact on people's working capacity and well-being [2].

Phacoemulsification, the standard surgical removal of the opacified lens and intraocular lens (IOL) implantation, is the only way for effective treatment. Phacoemulsification preserves the capsular bag formed by the anterior and posterior capsule, into which the IOL is implanted. Created in situ capsular bag allows light to pass freely through the transparent artificial lens and the thin acellular posterior capsule.

One of the most common late complications of this procedure is posterior capsule opacification (PCO), which develops from residual lens epithelial cell (LEC) proliferation and migration onto the posterior capsule. PCO can develop from weeks to several years after surgery, and depending on patient factors, IOL design, and surgical technique, it occurs in up to 50% of cases. It leads to decreased visual acuity and contrast sensitivity due to light scatter, remaining as one of the most significant challenges facing modern cataract surgery and healthcare systems [3,4].

The standard treatment for PCO is Nd:YAG laser capsulotomy, which uses focused laser energy to break down the fibrous tissue [5]. Although generally effective and non-invasive, it carries risks such as IOL damage, increased intraocular pressure, macular edema, and, in rare cases, retinal detachment [3,4].

In view of the above, efforts have been made to prevent PCO through modifications in IOL design, pharmacological strategies, and surgical techniques [6]. One such preventive measure is posterior capsulorhexis, a technique that involves the intentional removal of the central posterior capsule during surgery. This approach eliminates the substrate for LECs proliferation and may reduce the incidence of PCO and its associated complications [7]. However, the American Academy of Ophthalmology (AAO) does not generally recommend performing posterior capsulorhexis in patients above 8 years of age due to the increased risk of lens dislocation and potential weakness of the ciliary system.

In this case report, we describe the outcomes of a patient with bilateral congenital posterior subcapsular cataracts who underwent phacoemulsification with IOL implantation. One eye was treated with sthe tandard technique, while the other included intraoperative posterior capsulorhexis, performed using our novel technique. The markedly different postoperative courses illustrate the potential value of posterior capsulorhexis in preventing secondary complications.

## Case presentation

A 27-year-old female patient presented with complaints of visual impairment, specifically blurred vision and decreased distance visual acuity in both eyes. She had been wearing contact lenses and desired to discontinue their use. On objective examination, both eyes revealed high myopia, primary posterior subcapsular cataract, and astigmatism.

Snellen chart visometry revealed reduced vision in both eyes. In the right eye (VOD), uncorrected visual acuity was 0.5 (c/l), while in the left eye (VOS), it was 0.4 (c/l). Autorefraction confirmed high myopia bilaterally, with a spherical equivalent of -17.25 D in each eye. The right eye showed -16.75 D sphere with -1.00 D cylinder at 35°, and the left eye showed -17.00 D sphere with -0.50 D cylinder at 30°, indicating mild astigmatism (Table 1). Keratometric assessment revealed regular corneal curvatures, with mean keratometry values of 41.25 D (OD) and 41.00 D (OS), along with corneal astigmatism of -1.25 D at 15° in the right eye and -1.00 D at 160° in the left (Table 2). With the correction of -24.0 D sphere combined with -1.0 D cylinder at axis 35° in the right eye and -26.0 D sphere with -0.5 D cylinder at axis 30° in the left eye, the best-corrected visual acuity in both eyes improved to 0.5.

**Table 1:** Autorefraction (refractive error) before surgery Autorefraction revealed high myopia in both eyes, with a spherical equivalent of –17.25 diopters (D) in each eye. The right eye exhibited –16.75 D sphere with –1.00 D cylinder at 35°, and the left eye –17.00 D sphere with –0.50 D cylinder at 30°, indicating mild astigmatism bilaterally.

Eye	Sphere (D)	Cylinder (D)	Axis (°)	Spherical equivalent (D)
Right (OD)	–16.75	–1.00	35	–17.25
Left (OS)	–17.00	–0.50	30	–17.25

**Table 2:** Keratometry before surgery Keratometric measurements showed regular corneal curvatures, with mean keratometry values of 41.25 D in the right eye and 41.00 D in the left eye. Corneal astigmatism was –1.25 D at 15° in the right eye and –1.00 D at 160° in the left eye.

Eye	Flat K (D)	Steep K (D)	Mean K (D)	Corneal astigmatism (D)	Axis (°)	Corneal diameter (mm)
Right (OD)	40.50	41.75	41.25	–1.25	15	8.07–8.31
Left (OS)	40.50	41.50	41.00	–1.00	160	8.08–8.36

Tonometry using the Icare device revealed an intraocular pressure of 22 mmHg in the right eye (TOD) and 17 mmHg in the left eye (TOS). Biomicroscopic examination of both eyes showed normal eyelids and lashes, a clear conjunctiva, and a transparent, avascular cornea. The anterior chambers were of medium depth, and the pupils were round and centrally located. Primary cortical opacities were noted in the lenses, together with single opacities in the vitreous bodies. Specular microscopy confirmed preserved endothelial cell morphology, with normal cell density, polymegathism, and hexagonality, as well as stable corneal thickness values (Table 3).

**Table 3:** Corneal endothelial parameters assessed by specular microscopy (NIDEK CEM-530) before surgery Specular microscopy showed normal endothelial cell densities of 2971 cells/mm² (OD) and 3097 cells/mm² (OS), with average cell areas of 312 µm² and 282 µm², respectively. Coefficient of variation and hexagonality values were within normal limits, indicating good cell size uniformity and morphology. Central corneal thickness was 534 µm in the right eye and 540 µm in the left. Overall, findings suggest a healthy and stable corneal endothelium in both eyes.

Parameter	Right eye (OD)	Left eye (OS)	Reference range / interpretation
Number of cells analyzed (NUM)	93	104	≥100 recommended for reliable assessment
Cell density (CD, cells/mm²)	2971	3097	Normal: 2000–3000+
Average cell area (AVG, µm²)	312	282	Inversely proportional to cell density
Standard deviation (SD, µm²)	117	95	Indicates variation in cell size
Coefficient of variation (CV, %)	37	34	Normal: <40%; lower indicates homogeneity
Maximum cell area (MAX, µm²)	1053	1612	Wide ranges may indicate polymegethism
Minimum cell area (MIN, µm²)	136	124	
Hexagonality (HEX, %)	60	55	Normal: >50%; reflects cellular uniformity
Central corneal thickness (CT, µm)	534	540	Normal: ~500–560 µm

Fundus examination of the right eye (OD) revealed a pinkish optic nerve head with myopic conus and a normal macular reflex. Areas of chorioretinal atrophy were observed, along with argon laser coagulation scars in the superior, temporal, and nasal sectors. The choroidal blood vessels were clearly visible. In the left eye (OS), the optic nerve also appeared pinkish with myopic conus, and the macular reflex was preserved. Similar areas of chorioretinal atrophy were present, accompanied by argon laser coagulation scars distributed circumferentially (360°), which had been performed prophylactically due to peripheral retinal thinning and the increased risk of retinal detachment associated with high myopia. Choroidal blood vessels were likewise visible.

Optical coherence tomography (OCT) confirmed these findings, showing increased echogenicity of the pigment epithelium in the foveal area of both eyes, thickening of the overlying neurosensory layer, and poorly differentiated retinal borders due to lens opacity, consistent with myopic retinopathy.

**Figure 1:**
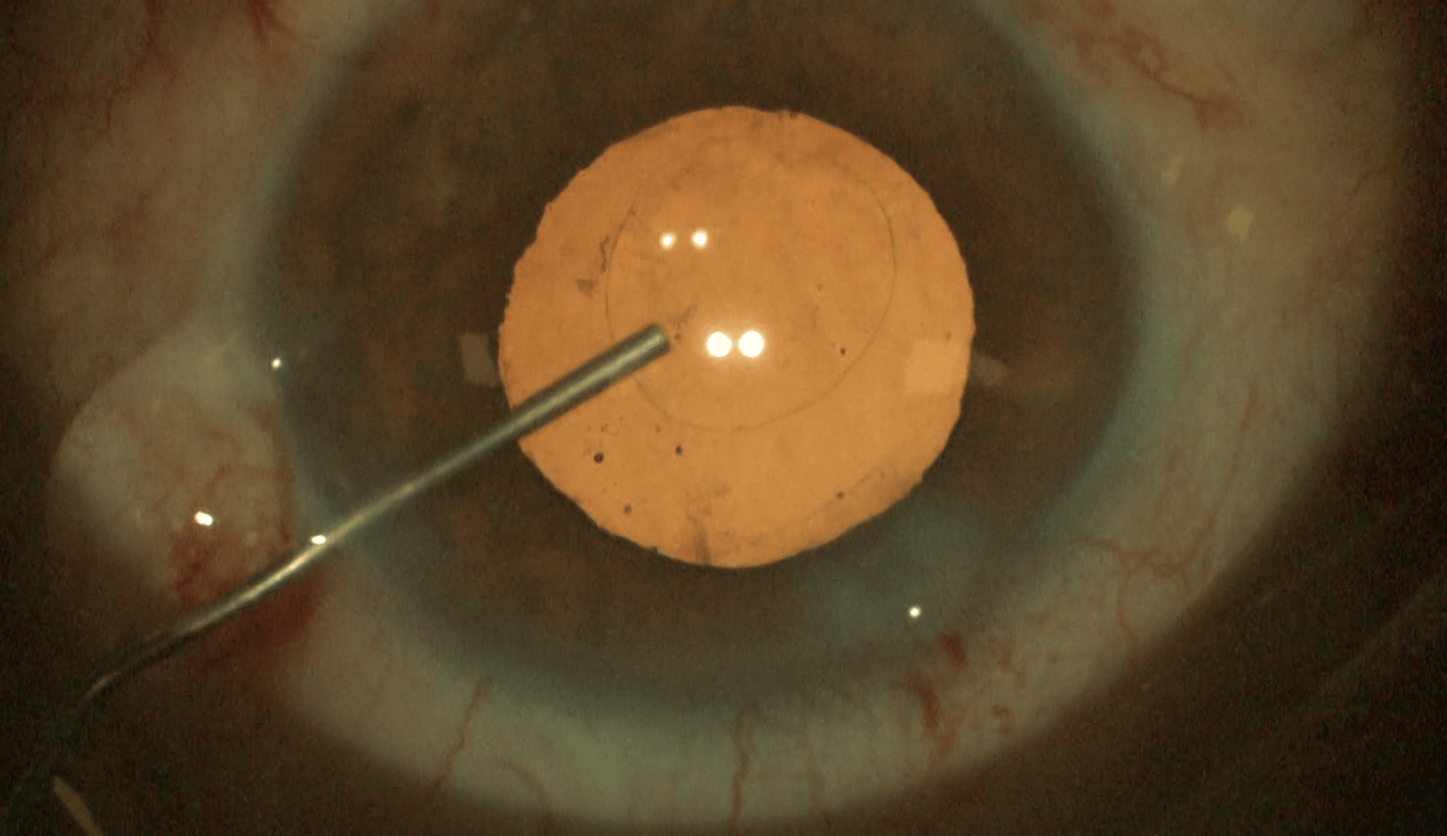
Intraoperative capsulorhexis

These findings indicated severe refractive error consistent with high myopia and mild regular astigmatism. Phacoemulsification and toric multifocal intraocular lens implantation were performed in both eyes for the combined treatment of congenital cataract, myopia, and astigmatism. In addition, an intraoperative posterior capsulorhexis technique was used in the right eye (Figure 1). Our novel posterior capsulorhexis technique differs from previously described methods and offers several advantages. After phacoemulsification and cortical aspiration, cohesive viscoelastic is introduced into both the anterior and posterior chambers to stabilize the capsule. The central posterior capsule is then punctured and lifted from the anterior hyaloid, followed by careful removal with capsule forceps in a continuous circular motion, minimizing traction on vitreous fibers. The capsular bag is simultaneously prepared for intraocular lens implantation, and the posterior capsulorhexis diameter is kept slightly smaller than the anterior opening to ensure stability and centration of the IOL.

One month after surgery, the visual outcomes were satisfactory. Snellen visometry demonstrated a visual acuity of 1.0 in the right eye (VOD). In the left eye (VOS), uncorrected visual acuity was 0.7, which improved to 1.0 with the correction of +0.25 diopters sphere and -0.5 diopters cylinder at axis 5°.

Intraocular pressure measured by Icare tonometry was 16 mmHg in the right eye (TOD) and 13 mmHg in the left eye (TOS), remaining within normal limits. Data from Tables 4-6 further confirmed stable postoperative results: autorefraction revealed only minimal refractive error (-0.25 D OD, +0.25 D OS), keratometry showed regular corneal curvatures with mild astigmatism (-1.25 D OD, -1.50 D OS), and specular microscopy demonstrated preserved endothelial cell density (2618 OD, 2492 OS) and morphology, with normal central corneal thickness (558 µm OD, 562 µm OS).

**Table 4:** Autorefraction (refractive error) in one month after surgery Autorefraction revealed minimal refractive error in both eyes, with a spherical equivalent of –0.25 D in the right eye and +0.25 D in the left.

Eye	Sphere (D)	Cylinder (D)	Axis (°)	Spherical Equivalent (D)
Right (OD)	0.00	–0.25	167	–0.25
Left (OS)	+0.25	–0.25	3	+0.25

**Table 5:** Keratometry in one month after surgery Keratometry showed regular corneal curvatures, with mean keratometric readings of 41.50 D (OD) and 41.25 D (OS). Corneal astigmatism was –1.25 D in the right eye and –1.50 D in the left, with axes at 13° and 169°, respectively.

Eye	Flat K (D)	Steep K (D)	Mean K (D)	Corneal astigmatism (D)	Axis (°)	Corneal diameter (mm)
Right (OD)	40.75	42.00	41.50	–1.25	13	8.30–8.32
Left (OS)	40.50	42.00	41.25	–1.50	169	8.26–8.32

**Table 6:** Corneal endothelial parameters assessed by specular microscopy (NIDEK CEM-530) one month after surgery One month after surgery, specular microscopy revealed endothelial cell densities of 2618 cells/mm² (OD) and 2492 cells/mm² (OS), with average cell areas of 360 µm² and 377 µm², respectively. Cell size variation remained within normal limits, with coefficients of variation of 36% and 37%, and hexagonality values of 71% (OD) and 67% (OS), indicating preserved endothelial morphology. Central corneal thickness measured 558 µm and 562 µm in the right and left eyes, respectively. Overall, the corneal endothelium showed stable postoperative status with no significant endothelial cell loss or morphological decompensation.

Parameter	Right eye (OD)	Left eye (OS)	Reference range / interpretation
Number of cells analyzed (NUM)	192	177	≥100 recommended for reliable assessment
Cell density (CD, cells/mm²)	2618	2492	Normal: 2000–3000+; slightly reduced post-op
Average cell area (AVG, µm²)	360	377	Inversely related to cell density
Standard deviation (SD, µm²)	129	141	Indicates variation in cell size
Coefficient of variation (CV, %)	36	37	Normal: <40%; indicates acceptable uniformity
Maximum cell area (MAX, µm²)	925	955	Range of cell sizes — within expected limits
Minimum cell area (MIN, µm²)	130	122	
Hexagonality (HEX, %)	71	67	Normal: >50%; reflects healthy morphology
Central corneal thickness (CT, µm)	558	562	Normal: ~500–560 µm; mildly elevated
Fixation quality (FIX)	CR	C	Good (C: centered, CR: centered/reliable)

Four months after the operation, the patient approached a regional clinic with decreased vision in the left eye. Examination revealed a secondary cataract due to PCO, and the patient underwent Nd:YAG laser posterior capsulotomy.

**Figure 2:**
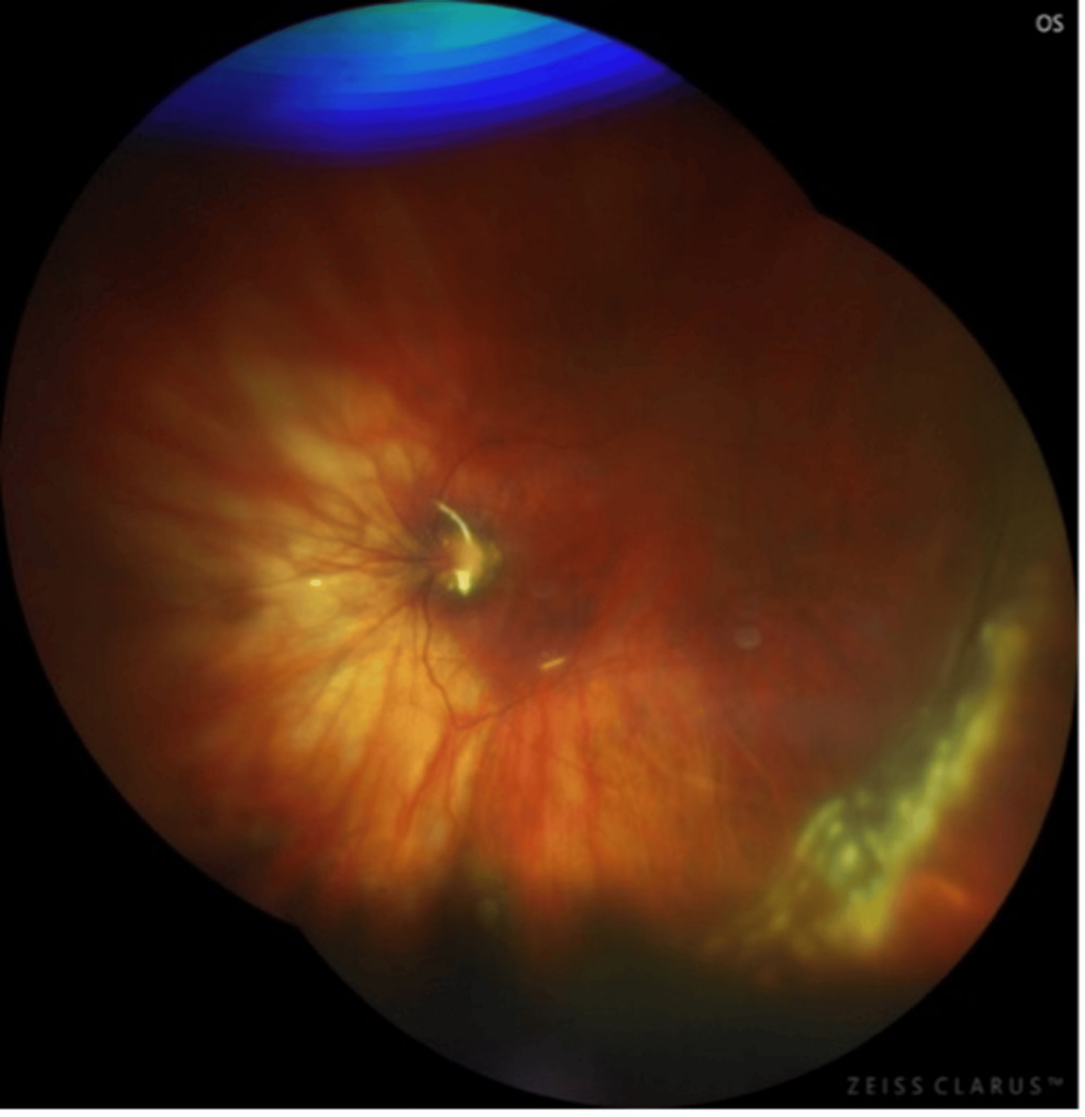
Retinal detachment in the left eye

Six days later, she referred to us with decreased vision and the presence of a shadow in the left eye. After examination, it was determined that there was a retinal detachment in the left eye (Figure 2). Vitrectomy was immediately performed.

At this point, vision in the patient’s left eye is 0.6, while her right eye remains intact, with vision of 1.0.

## Discussion

This case presents a rare combination of bilateral congenital posterior subcapsular cataract, high myopia, and astigmatism in a young adult patient who had a desire to completely eliminate her pathological ophthalmic conditions. A combined surgical strategy was chosen using phacoemulsification and toric IOL implantation, with intraoperative posterior capsulorhexis performed only in the right eye. The different postoperative courses in the two eyes highlight the potential importance of this technique in preventing secondary complications.

PCO is the most common delayed complication of modern cataract surgery, particularly in younger patients and in those receiving multifocal IOLs. It develops from the proliferation and migration of residual LEC onto the posterior capsule [8]. In this case, the left eye, which did not undergo posterior capsulorhexis, developed visually significant PCO within four months, making Nd:YAG laser posterior capsulotomy necessary.

While Nd:YAG laser capsulotomy is considered a gold standard for PCO treatment, it also causes numerous complications. On one hand, high myopia increases the risk of retinal detachment. On the other hand, this risk is further compounded by vitreous instability caused by Nd:YAG laser capsulotomy [9]. In this patient, the left eye developed a retinal detachment shortly after YAG treatment, requiring urgent vitrectomy. Visual acuity in that eye remains limited (0.6), underscoring the severity of such complications.

By contrast, the right eye, which underwent intraoperative posterior capsulorhexis, has remained clear and visually stable (1.0 vision) without the need for additional intervention. By removing the central posterior capsule at the time of surgery, the substrate for epithelial cell migration and fibrotic proliferation is eliminated. Although this technique is more commonly used in pediatric cataract surgery or in eyes with a high risk of PCO, this case supports its potential value in adult patients with risk factors such as young age, congenital cataract, high myopia, or implantation of multifocal IOLs. While the AAO recommends avoiding P-CCC in children above 8 years because of the generally lower risk of PCO and the importance of preserving capsule integrity for long-term IOL stability, in selected cases a technically skilled surgeon may still consider it beneficial. In such situations, careful patient selection and surgical expertise can help reduce the need for secondary Nd:YAG capsulotomy and its possible complications.

Limitations and clinical implications

Although this is a single-patient case report, the markedly different outcomes between the two eyes provide a promising argument for the use of posterior capsulorhexis in adult patients. This technique may reduce the need for Nd:YAG capsulotomy and its associated complications, particularly in high-risk individuals, such as those with congenital cataract and high myopia, younger adult age, or those receiving multifocal intraocular lenses. Larger studies are warranted to assess the broader applicability and safety of this technique in adult cataract surgery. 

## Conclusions

This case illustrates the potential benefits of considering intraoperative posterior capsulorhexis in cataract surgery, particularly in patients at high risk for PCO. While both eyes initially achieved satisfying visual outcomes, the eye that underwent standard phacoemulsification without posterior capsulorhexis developed visually significant PCO, ultimately complicated by retinal detachment after Nd:YAG capsulotomy. In contrast, the eye treated with posterior capsulorhexis remained free of opacification and complications, maintaining a stable visual acuity of 1.0. These findings support the consideration of posterior capsulorhexis as a preventive surgical strategy in adult patients - especially those with late presentations of congenital cataract or other high-risk features, where the surgeon is confident in their technical skills and employs meticulous, precise techniques, to safely reduce the risk of secondary interventions and sight-threatening complications. We believe that our novel technique can be further refined, and we hope that broader research will help establish its safety, efficacy, and wider applicability.
